# Design of a Multi-Control Objective Rescue Mechanical Ventilation System (Linshomator)

**DOI:** 10.3389/fmedt.2020.575964

**Published:** 2020-11-17

**Authors:** Aritra Roy, Pushpak Singh, Simran Saha, Arvil Sen, Mayukh Ray, Samarpan Deb Majumder, Sagnik Dutta, Shubho Chowdhuri, Raghav Mundhra

**Affiliations:** ^1^Department of Electronics and Communication Engineering, Institute of Engineering & Management, Kolkata, India; ^2^Department of Mechanical Engineering, Institute of Engineering & Management, Kolkata, India; ^3^Department of Information Technology, Institute of Engineering & Management, Kolkata, India; ^4^Department of Electrical Engineering, Institute of Engineering & Management, Kolkata, India; ^5^Murshidabad Medical College and Hospitals, West Bengal, India; ^6^Department of Mechanical Engineering, Indian Institute of Technology, Kharagpur, India

**Keywords:** ventilation, modes, discharge, maintainability, reliability

## Abstract

In recent years, the need for a low-cost emergency ventilation system has increased unprecedentedly. Mechanical ventilation systems are widely used to cater to sudden oxygen demands, low breathing rates, and critical conditions during bradycardia and tachycardia. In this research, a new design of mechanical ventilation system synced with the tidal volume requirements of the patient using a piezoelectric belt has been proposed. The device proposed has various modes of ventilation—ACV (assisted controlled ventilation), SIMV (synchronized intermittent mandatory ventilation), and NIV (non-invasive ventilation), depending on the patient's requirements. A digital interface or user-friendly software has also been developed to help medical professionals easily monitor a patient's medical conditions. Finally, the automation that controls the ventilation mechanism of the device has been tested and validated with a conventional ventilator, and it has been found that the accuracy of the device in terms of delivering the exact quantity of air into the patient according to his requirements has been improved significantly. Further, the comparative study of the experimental data indicated that 5–10% error in detecting inhale and exhale attempt of a patient was detected with the conventional ventilator.

## Introduction

The mechanical ventilation system provides a required volume of air at certain intervals during specific medical critical conditions. The tidal volume required for the ventilation, however, depends on certain parameters like breathing rate, pulse rate, oxygen levels, CO_2_ levels, gaseous mixtures, activities the patient is performing at a particular period, and so on. Modern ventilators are dynamic systems whose dynamic behavior and outputs are taken as signals in the in-built automated monitoring systems, and the ventilation at required amounts is then fed into the patient—this has ensured the decrease in manual monitoring of patients round-the-clock as well. Over the years of research, it can be inferred that in most of the mechanical ventilation systems, different parameters were used or integrated with automation to maintain its optimal performance. In a research work by Vallverdu et al. (1), the authors had presented a real-time study of the different clinical parameters involved in weaning during mechanical ventilation. The purpose of the research was also to find out which patients are able to maintain spontaneous breathing rates for a specific period of time and those who are unable to do so.

Based on the establishment of different clinical parameters affecting the ventilation system, many portable or low-cost ventilation devices were proposed and built, but not every device accounted for all the parameters. In the patent work by Masic (2), he had developed a ventilation system based on the relationship between measurable pressure and measurable flow of a patient to determine the requirements of ventilation. In the patent, the inventor has also taken into account some peculiar instances where the relation between, inter alia, measured pressure, measured flow, and patient effort was considered and linear regression techniques were used to reckon the patient efforts. In another patent work by Bonassa (3), the inventor developed a ventilation system that possesses a respiratory circuit with re-inhalation for the administration of medical problems like anesthesia. The ventilation system developed by the inventor comprises of bellows assembled within a reservoir integrated with various valves for proper gaseous exchange, controlling the gas mixtures and to release excess gas. The device was also given additional oxygen supply lines to compensate for any chances of shortage of oxygen. In a similar patent work by De Vries et al. (4), the inventors presented an invention that comprises a portable ventilator that uses a small, low-inertia, high-speed, high-efficiency ROOTS-type blower in variable-speed mode to reduce the noise created by the ventilation system, which might cause uncomfortable situations for patients who are in need for round-the-clock assistance or monitoring.

It is quite worthy to note that in a research work by Basra et al. (5), not only the cost of the ventilation system is reduced as whole, but a fundamental technique for controlling the breathing patterns was also used—which is a critical part of ventilation. This paper has presented that with the required amount of breathing rates per minute, we can control the required amount of tidal volume of air to be fed into the patient. The breathing pattern here is mapped based on the temperature rise during exhale cycles. In the proposed ventilation system, almost all the medical parameters have been taken into account by dividing the mode of operation of the system into two—manual and automated. In the automated mode of the ventilation system, the breathing rate per minute has been synced with the servo motor, which shall control the given amount of strokes required per minute. The strokes indicate one complete gush of 350-ml capacity of air into the patient. The automated mode also consists of the recording of beats per minute and oxygen level requirements with the sensors used, which will be displayed in the LCD screens. The manual mode shall include the monitoring of the doctor or the person in charge of the patient. As the oxygen level and beats per minute would be shown on the screens, the oxygen supply lines shall be controlled or manually opened at the desired levels.

This research paper also presents the real-time breathing analysis of 10 patients and also how the automation system has been synced with the inhale and exhale attempts of the patient to ensure proper ventilation. The automation module has been discussed in detail in further sections of this manuscript and a detailed study of its synchronization with the mechanical ventilation system has also been presented. Also, since the coronavirus releases proteins that attach themselves to the ACE2 receptors in the lungs of a human body as mentioned in the research work by Menter et al. (6), and ultimately resulting in breathing problems, an efficient ventilator device shall be extremely beneficial. As the proposed design is portable and lightweight and efficient in terms of air delivery, this device can therefore be used both during the COVID-19 pandemic and post-pandemic.

## Methodology

This section of the manuscript describes the various features of the ventilator device such as the design, automation involved in maintaining the optimal functioning of the device, and simulations of air flow and thermal profile.

### Design of the System

**Figure 1:**
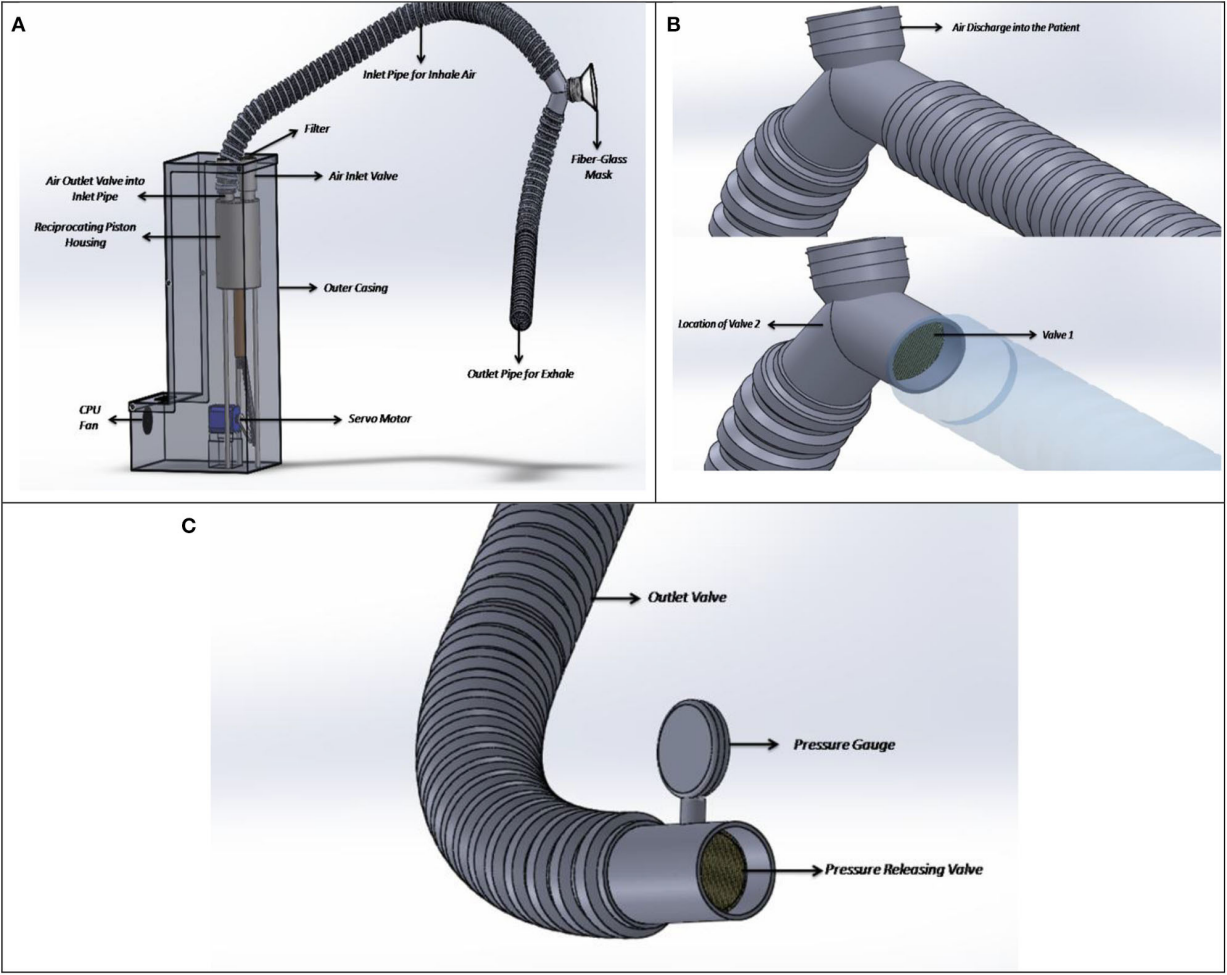
**(A)** Proposed design, **(B)** check valves installed at the patient air intake and exhale points, and **(C)** pressure-releasing valve and gauge.

The mechanical ventilation system is the one that provides a required volume of air at certain intervals during specific medical critical conditions. The tidal volume required for the ventilation, however, depends on certain parameters like breathing rate, pulse rate, oxygen levels, CO_2_ levels, gaseous mixtures, and activities the patient is performing at a particular period and so on. The proposed design of multi-purpose mechanical ventilation system is shown in Figure 1. In this figure, the mechanical ventilation mechanism is demonstrated where we are using a 1.05-L volume displacement reciprocating pump and a smaller size servo motor having a total torque of 1.37 N-m. The servo motor has been selected due to the requirements of different angles of rotation rpm for meeting different air discharge requirements. The maximum displacement achieved can be 1.05 L (1,050 ml), which is directly proportional to 1.05 L of air displacement or the required amount of air fed into the patient. In the proposed design, in one full stroke, the system can deliver 1,050 ml of air to the patient. For delivering a full stroke, the servo has to rotate 180°. A clearance volume of 0.05 L has been considered to avoid the piston touching the upper portion of the piston chamber. So, to determine the extent of rotation to deliver a fixed volume of air, we use Equation 1,


(1) $$\begin{array}{r} \text{rotate\_angle}=\frac{\text{tidal\_volume}}{1,050}\times 180 \end{array}$$

Now, the motor gives the required push continuously to deliver the tidal volume and pauses to complete the inhale time. After delivering the set tidal volume (7), it again returns to its initial position and wait for further instruction. For developing the air delivery mechanism, two one-way valves have been selected to be installed at the inlet and outlet of the piston housing—one for air intake into the chamber and the other for air outlet into the inlet pipe attached to the patient's mask. The outer casing as shown in Figure 1A shall comprise of all the mechanically operated parts as well as the automation modules. This casing shall be made of polysulfone plastic, which not only is lower in density thereby lowering the weight of the total equipment but also has excellent heat-resistant properties, having a thermal conductivity of around 0.26 W/mK. To ensure that proper cooling is achieved in the equipment, a 12-V CPU fan shall be connected. The CPU fan shall induce proper ventilation through the vents as provided in the casing so that workable temperature can be maintained inside the device for safe working of the sensors. The filter of standard grade and size shall be used—HEPA type, which shall reduce the contamination inside the pipes by 99%. This shall not only ensure proper intake of fresh air through the pipes but will also eradicate the chances of bacterial infestation inside the reciprocating cylinders. In Figure 1B, the pipes along with the valves are shown. Here, check valve 1 shall ensure that proper rate of discharge is maintained in relation to the patient's breathing rate. During the air intake cycle, the inlet valve shall remain open and the exhaust valve shall remain closed for any given duration, and the same thing would be repeated for exhale cycles. During the exhale cycle, the exhale valve shall remain open while the inlet valve shall remain closed. Down the exhale pipe line, a pressure-releasing valve has been set, which would not open until a desired amount of pressure is generated. This pressure is known as the plateau pressure—which shall not allow the patient's lung to collapse due to a certain decrease in pressure. The pressure-releasing valve as shown in Figure 1C can be calibrated according to the patient's need or doctor's requirements. However, a nominal pressure of 0.025 bar (30–35 cm of H_2_O) is set in the pressure gauge.

### Automation

The automation system incorporated very cheap yet powerful devices to address the issues. The algorithm has been implemented in a unique way so that the ventilation can be done in proper ways, addressing and monitoring all major issues of ventilation. The algorithm also ensures that medical parameters are controlled preciously, and in case of any emergencies, there are proper alarm systems. This section has been divided into three subsections—Block Diagram, Schematic Explanation, and Algorithm Analysis.

#### Block Diagram Analysis

**Figure 2:**
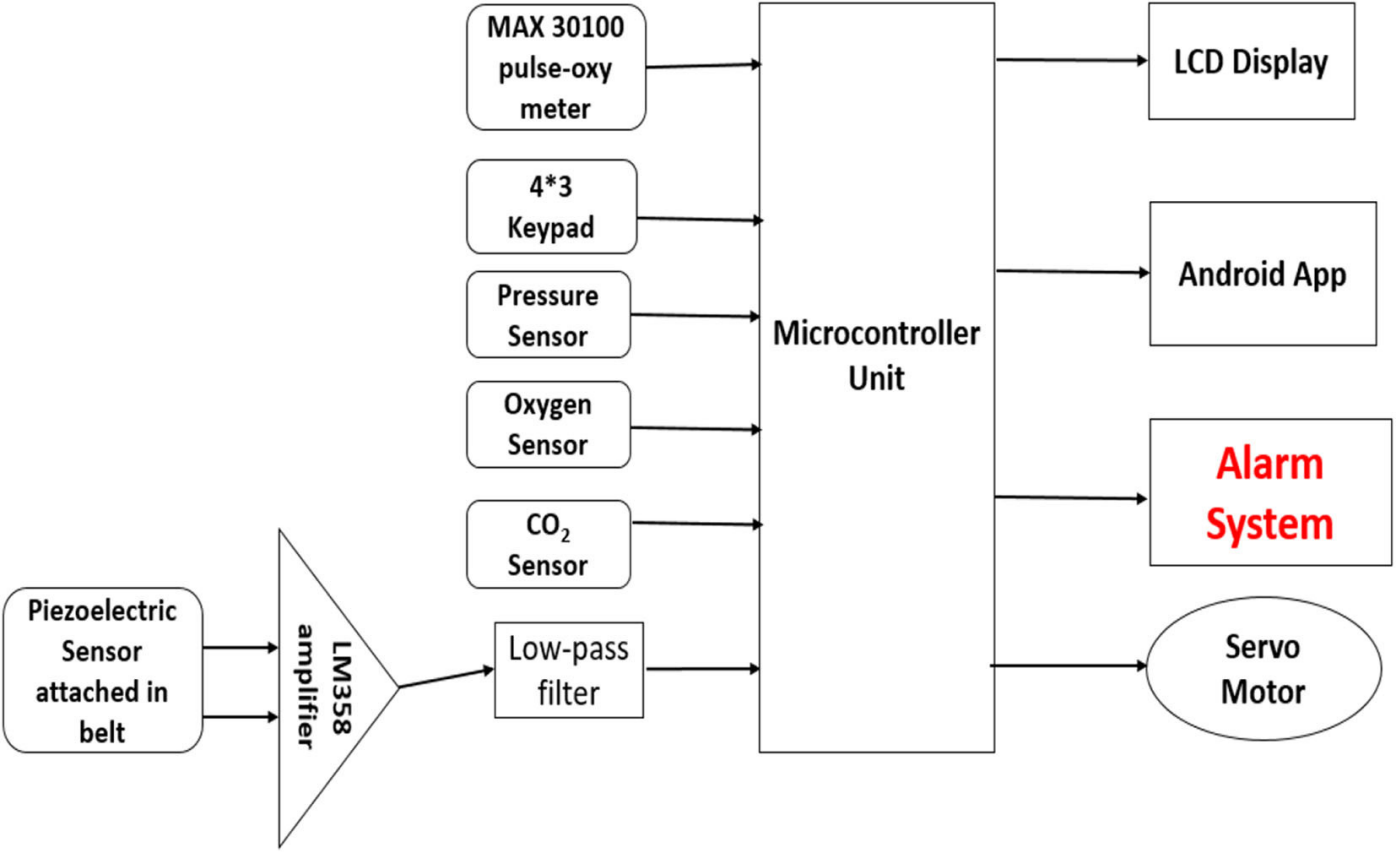
Block diagram of electrical system.

In the system as shown in Figure 2, a MAX30100 pulse-oxygen sensor is used for measuring the pulse rate and saturated oxygen percentage present in the blood of the patient. It combines two LEDs, a photo detector, optimized optics, and low-noise analog signal processing to detect pulse oximetry and heart-rate signals. The sensor can be attached to the patient's index or earlobe for measurements. The sensor will be interfaced with the microcontroller using I2C interfacing. For breathing pattern analysis and to monitor breathing rate, a belt containing a piezo-electric sensor was used, which would be wrapped around the chest of the patient. The piezo-electric sensor will detect the spikes when the lungs expand and, thus, measure the breathing pattern. The output of the sensor will be amplified using LM358 IC and then it will be passed through a RC low-pass filter before feeding it to the analog pin of the microcontroller. The MCU analyzes the change in voltage level and detects whether an inhalation is started by the patient. For taking input from the user for various manual operations, a 4^*^3 keypad is used and the data are fed to the microcontroller unit for processing. For the processing part, Arduino Uno, an open-source microcontroller board controlled by Atmega 328P (AVR 8 bit), was incorporated in the design. Although Arduino Uno was used for design prototyping, more advanced microcontrollers with better clock speeds and response times can also be used, as per system requirements.

It has 32 kB of flash memory and runs with 16 MHz clock speed. The inputs from various sensors are fed to this MCU, and after processing the data, it gives the necessary instruction to various output devices and also to the sensors. A 20^*^4 LCD to display the proper instruction processed by MCU to the user was also installed. There will also be provision of adding an O_2_ and CO_2_ sensor to monitor the oxygen and carbon dioxide concentration in inhaled and exhaled air. A servo motor was used in the design to push the piston to deliver the proper amount of air. A servo motor is an electrical device that can push or rotate an object with great precision. Servos are controlled by sending an electrical pulse of variable width, or pulse width modulation (PWM), through the control wire. Here, the servo receives instruction from the MCU and rotates accordingly to deliver the required amount of air (tidal volume) through the piston movement. Moreover, an Android app was also designed to alternate the parameters and to display the required information in a user-friendly manner. To ensure the safety of this system, an alarm system was also incorporated in this design. If at any point there is any leakage or sudden increase in any parameters, the system will trigger the alarm. With the help of the Android app, the users and the concerned authorities can also be notified.

#### Schematic Analysis

**Figure 3:**
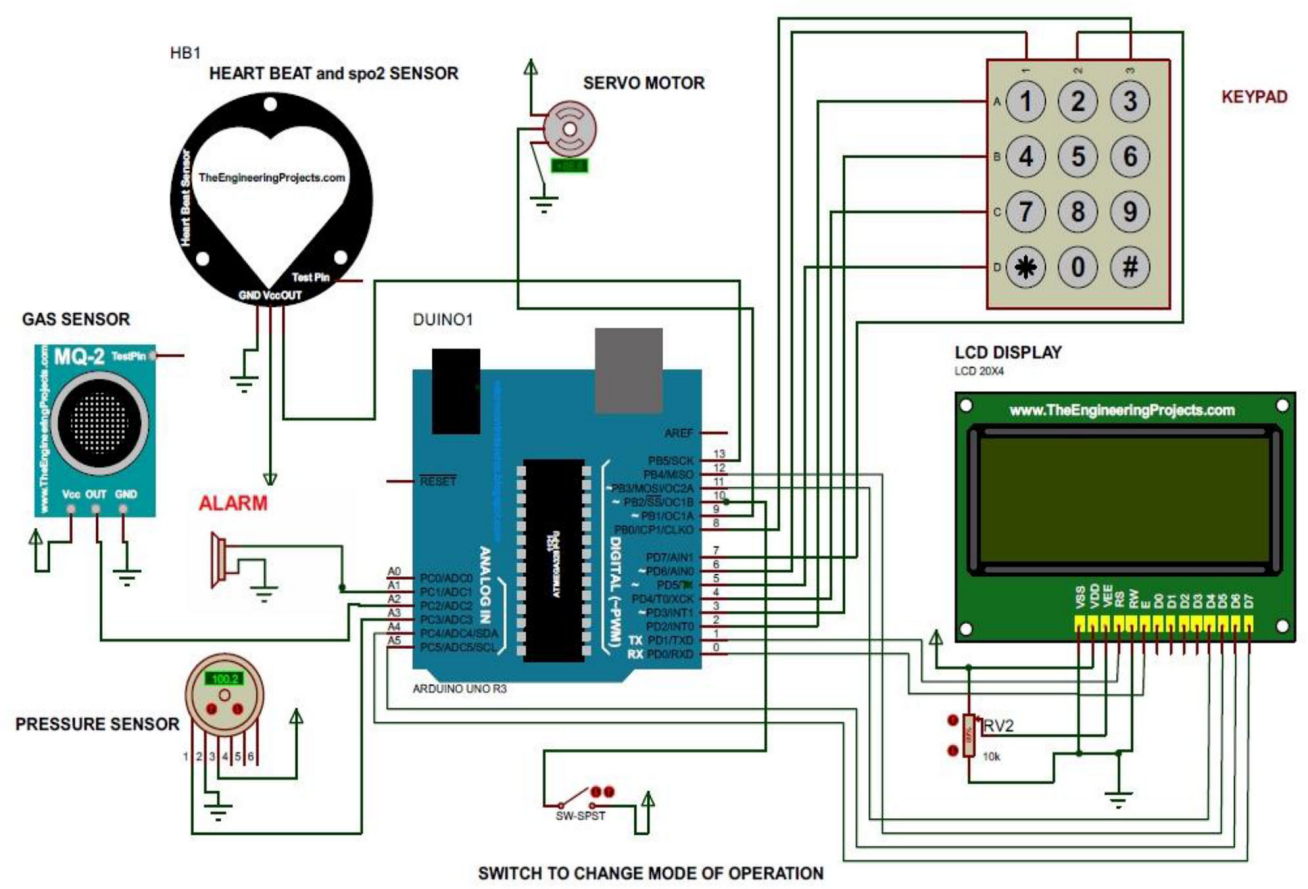
Complete circuit diagram of ventilation system.

In the system as shown in Figure 3, various sensors and display units along with the servo drivers are connected through the Arduino microcontroller using digital pins. Some of the sensors are interfaced with the MCU using I2C interfacing. An alarm system was also incorporated to ensure the safety.

**Figure 4:**
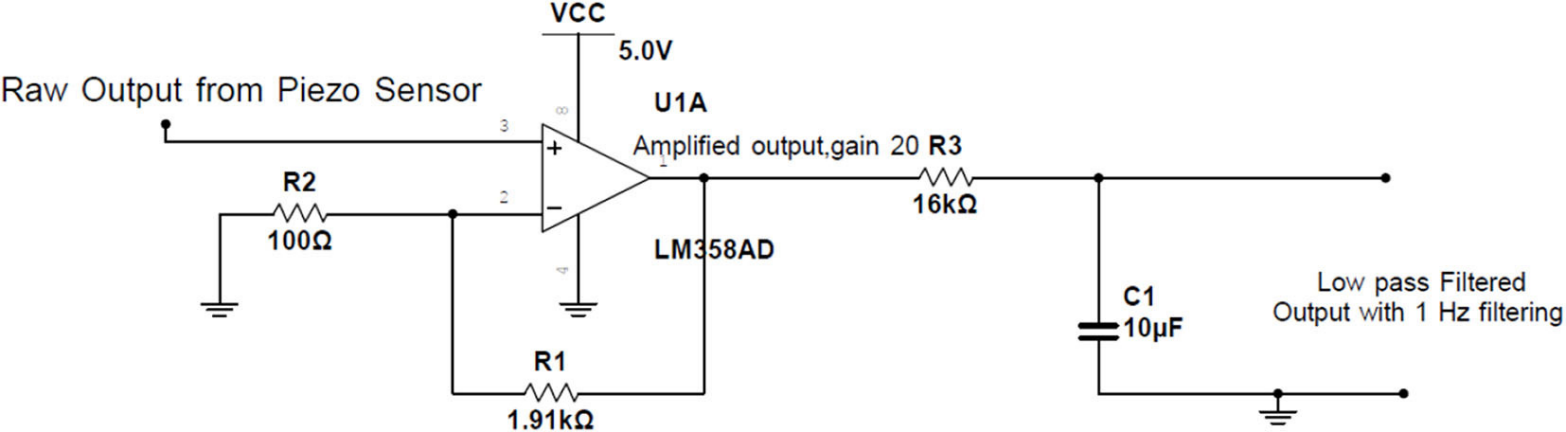
Amplifying circuit.

In the system, it was noticed that the change in voltage in the piezo sensor due to the expansion of the chest was very small. So, LM358 IC was used to amplify the signal with a gain of 20. The output is then filtered through a RC low-pass filter having R = 16 kΩ and C = 10 μF to obtain 1 Hz filtering as shown in Figure 4.

## Results and Discussion

This section of the manuscript deals with the discussion of the results obtained through the experimental runs with the developed equipment in the simulated environment.

### Algorithm Analysis

**Figure 5:**
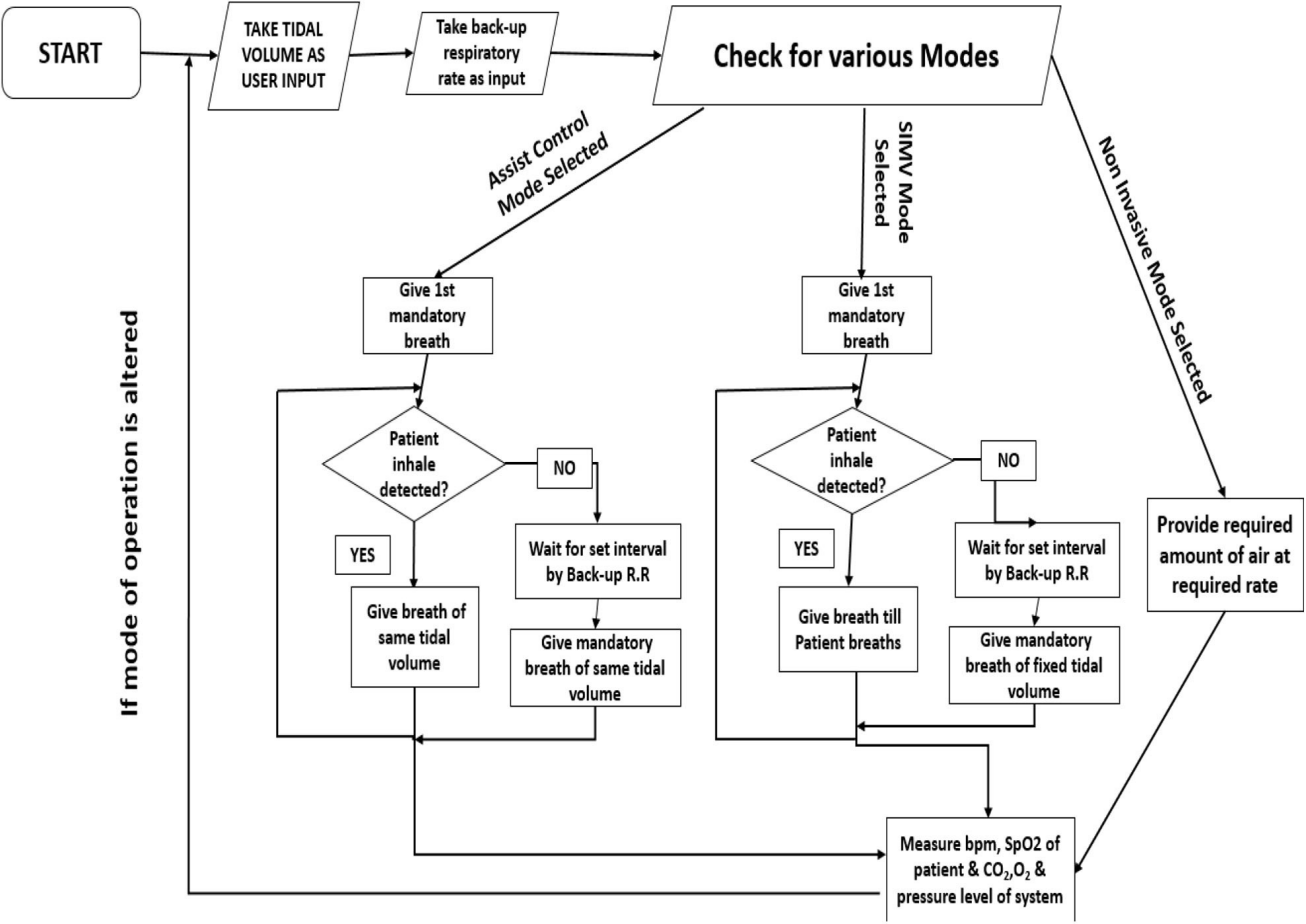
Algorithm layout.

In our system, we primarily have incorporated three modes of ventilation, assisted control mode of ventilation, synchronized intermittent mandatory ventilation (SIMV) mode, and non-invasive mode of ventilation. At first, as shown in Figure 5, the system will take the input from the user through the keypad or Android app about the tidal volume, backup respiratory rate, and I:E ratio. Now, the system will check for the modes of ventilation. If the AC mode is selected, it works in the following way. The system at first will deliver a mandatory breath. Now, until the occurrence of the next mandatory breath (time duration set as per backup respiratory rate), the system waits for the patient's effort to take a breath. As the inhale effort chest expansion is also associated, the microcontroller senses the changing voltage value from the piezo sensor, and if a spike is detected, it takes control and delivers air of a set tidal volume. Then, again, it returns to the mandatory breath. Now, if the mode is set to SIMV mode, the system performs in the same way, except the patient initiated breath. In that case, instead of fixed tidal volume, it gives continuous ventilation as per the patient's will with no fixed tidal volume.

**Figure 6:**
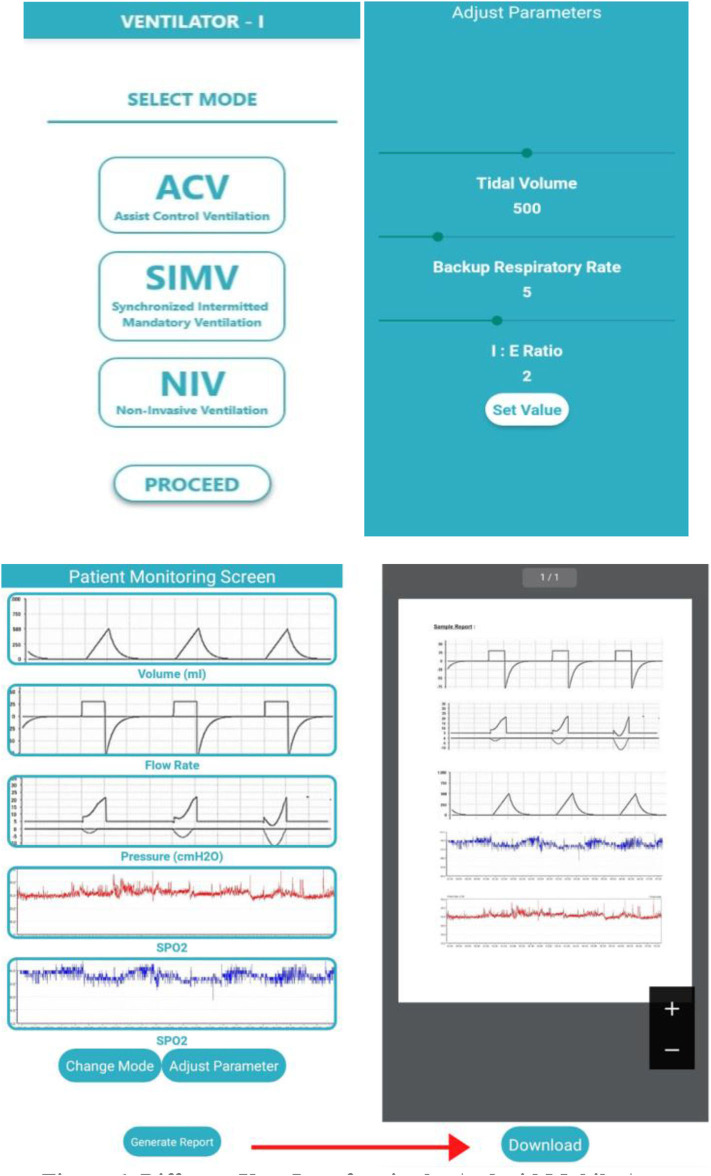
Different user interface in the Android mobile app.

If the mode is selected to non-invasive ventilation, the ventilator will give air of a fixed volume at a fixed rate with a fixed I:E ratio. The system will also monitor various parameters like breathing pattern, pressure in airways, and SpO_2_ and will continuously display the data in the Android app for monitoring (Figure 6). If the mode is changed or the parameters are altered, the control goes back to the beginning. The MCU will be fully integrated with the developed Android app through various connectivity options to add more user credibility to the design. As this system deals with very serious medical patients, some safety protocols were also incorporated in the design. At any point of operation, if there is any kind of sudden drop in system pressure (indicates leakage), or any sudden increase, it will set off an alarm system. Additionally, in case of any sudden changes in any kind of ventilation and medical parameters, similarly, alarm systems will be triggered. With the help of the proposed integrated Android app, the concerned authorities can be notified immediately in case of any emergencies.

In Figure 6, the different interfaces of the app have been illustrated. In the first interface, the patient or the person handling the app shall be provided with options to select for the mode of ventilation. Once the mode is selected, the different parameters can be measured and set at the required initial magnitudes as per the patient's requirements. The MCU shall then take over and set the parameters according to the patient's breathing rate. Thirdly, upon setting the values, a third window will open up where the monitoring screen shall display the different time-to-time fluctuations in the readings of different parameters. Lastly, the medical report containing the time-stepped medical results can be directly harnessed from the software and downloaded. This report can then be either shared virtually with a doctor or can be used as a reference to the patient's previous medical conditions during in-person appointments.

### Fluent Simulation of the Ventilation Pipes

This section discusses the design and simulation details of the project. Firstly, the geometry of the ventilator pipes is discussed. Then, the meshing induced for the simulation is discussed briefly, and then, the FLUENT flow simulation is demonstrated.

#### Flow Simulation

**Figure 7:**
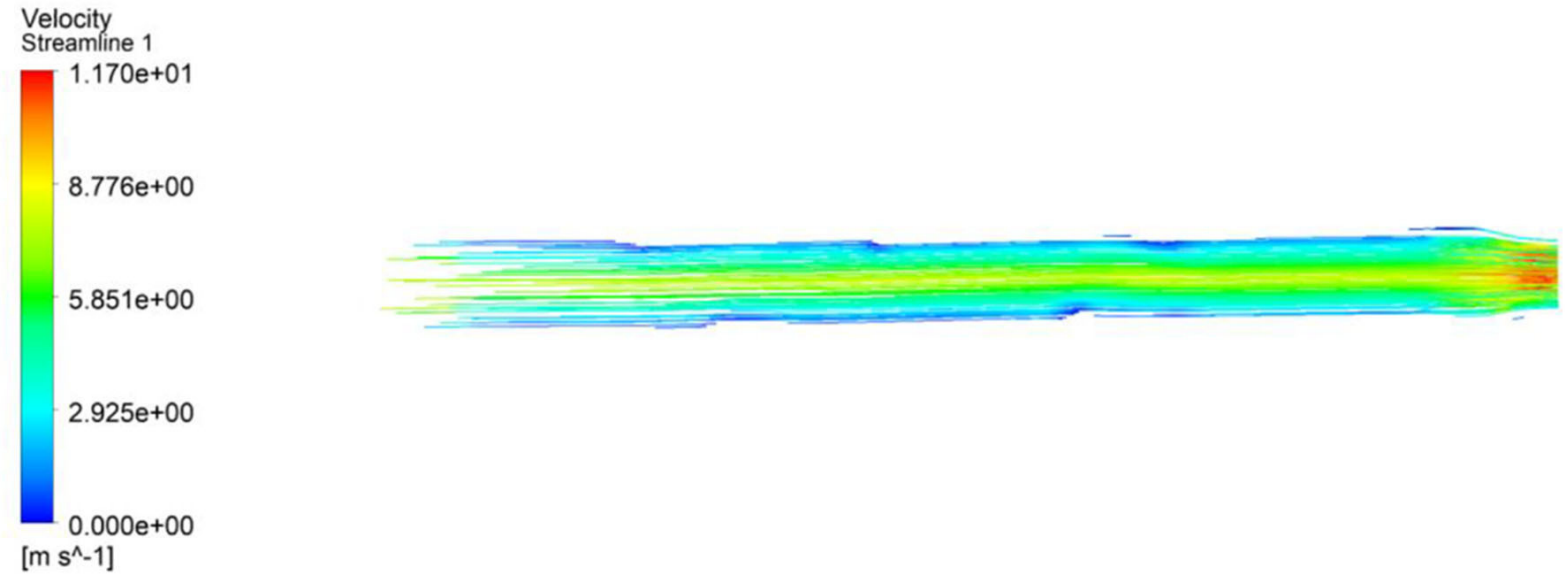
ANSYS simulation.

The flow simulation was studied to estimate the air flow velocity at the exit of the inhaler pipe of the ventilator. Although the velocity of air depends on the frequency of the servo motor, the air velocity can be altered according to the patient's requirements. From Equation 1, it can be inferred that with a given change in the angle of the servo shaft, the piston will deliver a given amount of air into the patient. To make sure that the air would be delivered uniformly, the flow simulation is an important step. The ventilator pipe has been designed with apparatus dimensions of (1) length = 1,000 mm, (2) larger circular cross-sectional diameter = 44 mm, and (3) smaller circular cross-sectional diameter = 36 mm. The design was done in the *Y*–*Z* axis of the ANSYS workbench. The proper selection of discretization of mesh is an important criterion for the achievement of the higher accuracy of the simulation results. The grid selection is therefore directly proportional to the simulation accuracy. For this study, hexahedral mesh of size 2.51e−3 was selected to obtain the optimal accuracy. At the inner and outer walls of the cylindrical pipe, the meshing was considered as refinement and the region of thickness at the inlet and outlet was considered to be tetrahedral of the automatic method. The grid independence was selected as mentioned due to the need of higher accuracy in the heat transfer through the cylinder walls and lesser amount of computational time. The flow setup was performed using the k-ε model and the experimental fluid as air. The entering velocity was taken as an input parameter of around 7.47 m/s. Here, the flow has been taken as turbulent flow inside the pipe with a Reynolds number of around 20,000. After undergoing 1,000 iterations, the velocity gradient as shown in Figure 7 was developed.

From the figure above, we can infer that the highest velocity is achieved at the outlet or at the discharge point of the ventilator pipes. The cross section being smaller than the previous one enables an increase in the velocity with which the air is fed into the patient, at around 12 m/s. From Figure 7, it can also be concluded that the air distribution is uniform throughout the pipe. As a no-slip condition has been considered, therefore, in some areas in the pipe, the air velocity is around 0 m/s at the vicinity of the wall. However, toward the end of the pipe, the velocity at the vicinity of pipe walls increases more than 0 m/s, thereby resulting in better air distribution through the mask attached with the pipe.

#### Thermal Analysis of the System

**Figure 8:**
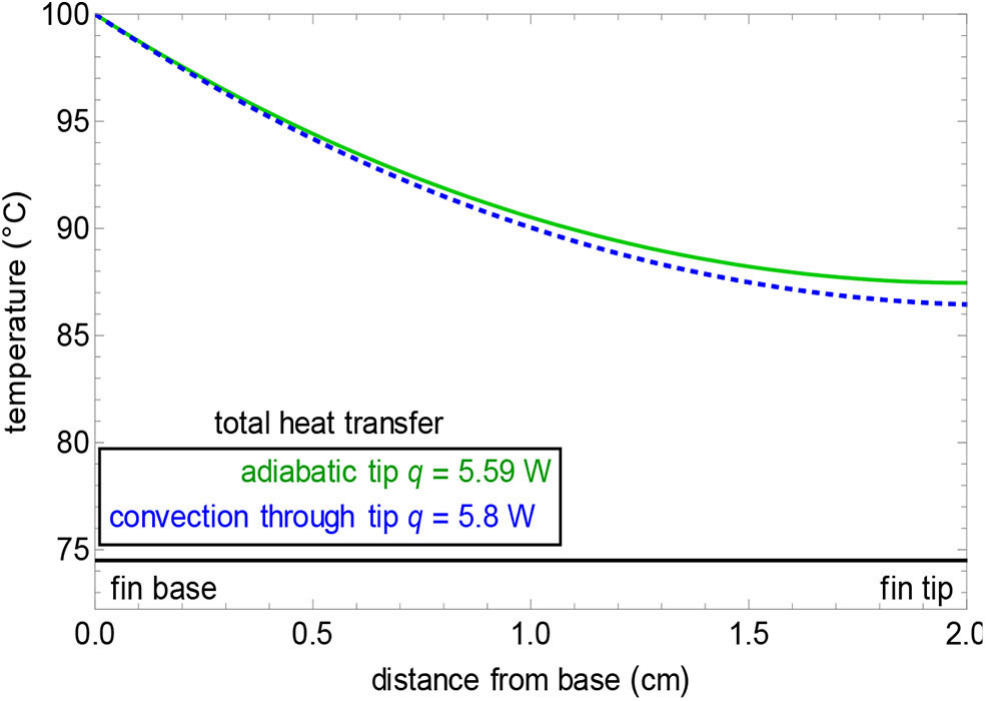
Graphical representation of the temperature profile of fin.

As the only component that shall undergo generation of heat is the motor, fins have been provided, which shall produce natural cooling through convection. During the simulation (shown in Figure 7), the base temperature of the motor has been considered to be 60–70°C, which is the maximum working temperature of a servo motor. The temperature rise will vary according to the work cycle the equipment has to sustain for periodic intervals. For the simulation, the material was specified as polysulfone with a thermal conductivity of around 0.26 W/mK. The heat transfer coefficient has been considered to be 0.5–1,000 W/m^2^K based on the velocity with which the air is flowing at. The average fin profile is shown in Figure 8 along with the graphical demonstration of the temperature profile with respect to the distance from the base to the tip. The governing equations (Equations 2–8) while computing the simulation are as follows:


(2) $$\begin{array}{r} {\text{T}}_{\text{adi}}=\frac{\cosh\left(\text{m}\left(\text{L }-\text{ x}\right)\right)}{\cosh\left(\text{mL}\right)}\left({\text{T}}_{\text{b}}-{\text{T}}_{\text{a}}\right)+{\text{T}}_{\text{a}} \end{array}$$

(3) $${\text{T}}_{\text{conv}}=\frac{\cosh\left(\text{m}\left(\text{L-x}\right)\right)\text{+}\frac{\text{h}}{\text{mk}}\sinh\left(\text{m}\left(\text{L-x}\right)\right)}{\cosh\left(\text{ml}\right)\text{+}\frac{\text{h}}{\text{mk}}\sinh\left(\text{mL}\right)}\left({\text{T}}_{\text{b}}-{\text{T}}_{\text{a}}\right)+{\text{T}}_{\text{a}}$$

(4) $$\begin{array}{r} \text{m}=\sqrt{\frac{\text{hP}}{\text{kA}}} \end{array}$$

(5) $$\begin{array}{r} \text{P}=2\text{w}+2\text{h} \end{array}$$

(6) $$\begin{array}{r} \text{A}=\text{wh} \end{array}$$

(7) $${\text{q}}_{\text{conv}}=\text{M}\frac{\sinh(\text{mL})\text{+}\frac{\text{h}}{\text{mk}}\cosh(\text{mL})}{\cosh(\text{mL})\text{+}\frac{\text{h}}{\text{mk}}\sinh(\text{mL})}$$

(8) $$\begin{array}{r} \text{M}=\sqrt{\text{hPKA}}\left({\text{T}}_{\text{b}}-{\text{T}}_{\text{a}}\right) \end{array}$$

where T_b_ and T_a_ are the base and ambient air temperatures, L is fin length (m), x is position down the fin (m), h = 50 W/(m^2^ K) is the convection heat transfer coefficient, k is the thermal conductivity of the material [W/(m K)], m is a simplification term, P is fin perimeter (m), A is the fin cross-sectional area (m^2^), w and h are the width and height of the rectangular fin (m), d is the pin fin diameter (m) and q is in W, and M is a simplification term. In **Figures 12**, **13**, the temperature profile can be understood as the hottest ones being the red zones and the coolest ones being the blue zones. The intermediate zones refer to the transition in temperature from hot to cold.

From Figure 8, it can be inferred that with single fin dimensions such as length = 2 cm, width = 0.2 cm, and height = 40 cm, we can achieve a temperature reduction of around 13°C. Now, if the number of fins increases, then the heat dissipation will be higher and we can achieve a base temperature in equivalence to that of the ambient temperature within a very short interval of time.

## Ergonomics of the Design

**Figure 9:**
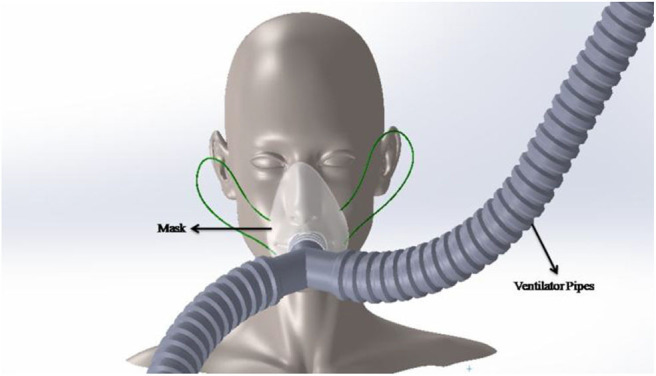
Human face setup.

The design has been done considering all the minute dimensions of the human face so that the mask fits perfectly, allowing the right quantity of air to be fed into the patient and maintaining optimal human comfort. Figure 9 demonstrates the face of a human where the mask has been installed. The mask as mentioned previously will be attached to the ventilator pipes.

**Figure 10:**
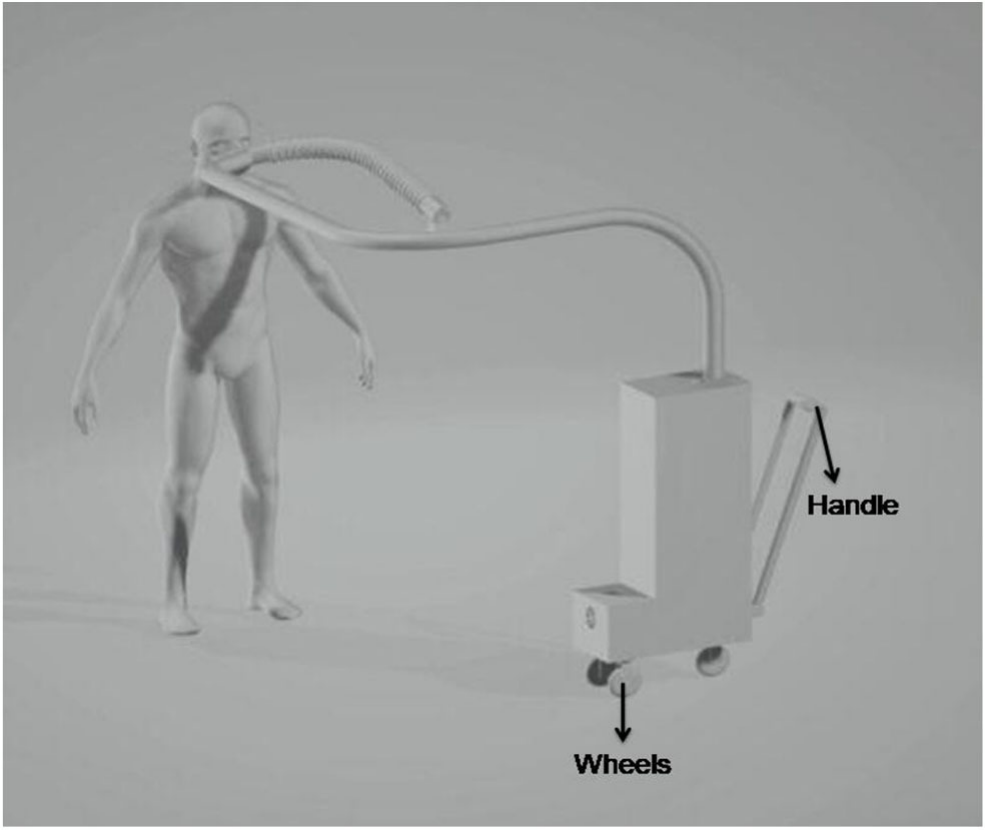
Linshomator setup with human.

The entire setup along with the entire human body as shown in Figure 10 has been designed in Solidworks workbench. For ease of portability, a handle and wheels have been attached to the device so that it can be transported from one location to the other at any given point of time.

## Validation

**Figure 11:**
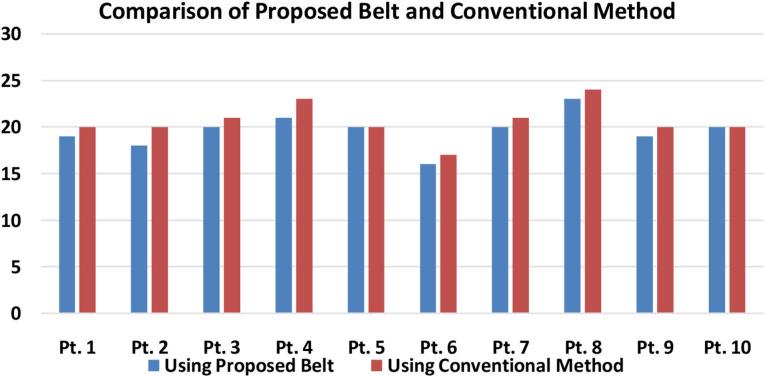
Comparison of breathing rate obtained from the proposed piezo belt and from the conventional method (Pt, patient).

The real-time breathing rate data were extracted and measured from 10 patients from Murshidabad Medical College and Hospital under the supervision of Dr. Shubho Chowdhuri using the developed piezoelectric breath rate monitoring system as shown in Figure 11. To compare the proposed design's efficiency, the measurement of the breathing rate of the patients was also taken with the help of a conventional ventilator apparatus. The patients examined had mediocre to severe breathing problems. It is important to note that coronavirus-affected individuals were not examined with the device, but due to the variety of patients examined, it can be concluded that the device would be suitable for all types of breathing deficiencies.

Figure 11 shows a typical comparison of the breathing rate control of the patients using the conventional ventilator and the proposed automated ventilator device. It can be well-understood that for almost all the patients, when the proposed system is used, the error in detecting the actual breathing rate of the patients has been lowered. For example, in the case of patient 1, the breathing rate measured using the conventional apparatus was around 20 bpm, whereas the piezoelectric belt, which is a more sensitive device (8), showed a reading of around 19 bpm. The same applies to other patients as well, and therefore, the error found in the readings can be approximated to around 5–10%. This indicates that the ventilator is able to properly detect the patient's breathing rate and deliver the exact required amount of air into the patient. Therefore, the proposed system has optimal accuracy in detecting the inhale and exhale attempt and can initiate the process of ventilation with a negligible delay.

**Figure 12:**
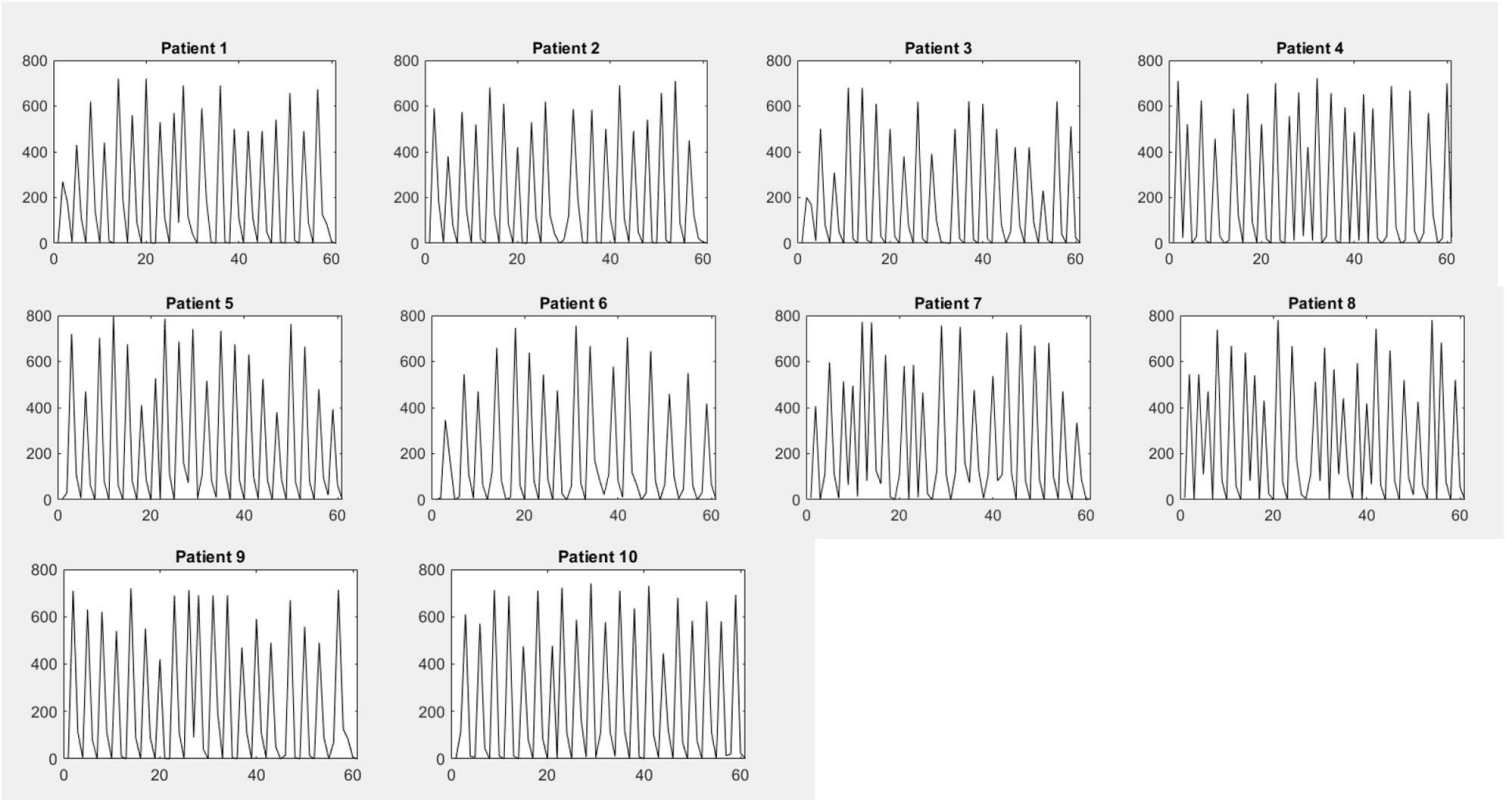
Analog readings obtained from patients 1–10 in a 60-s time frame.

Figure 12 shows the variation of analog reading obtained from 10 patients with respect to time to sense the inhale and exhale, where the *x* axis represents time frame and the *y* axis represents analog readings obtained from sensors. The voltage level of 0–5 V was mapped to 0–1,023 in the DAC present in the Arduino microcontroller. The peaks occurred due to the inhale attempt of the patient. Number of peaks occurring in a 60-s time frame corresponds to the breathing rate of the patient. The electrical circuits described in Figures 3, 4 were simulated using Proteus Software. The simulation gave satisfactory results in terms of proper rotation of servo motor to deliver the required amount of tidal volume. Besides, it was also able to detect the inhale and exhale attempt accurately. An emergency scenario was also simulated, and the alarming function also worked properly. The automation execution can be viewed in the video uploaded along with this manuscript.

**Figure 13:**
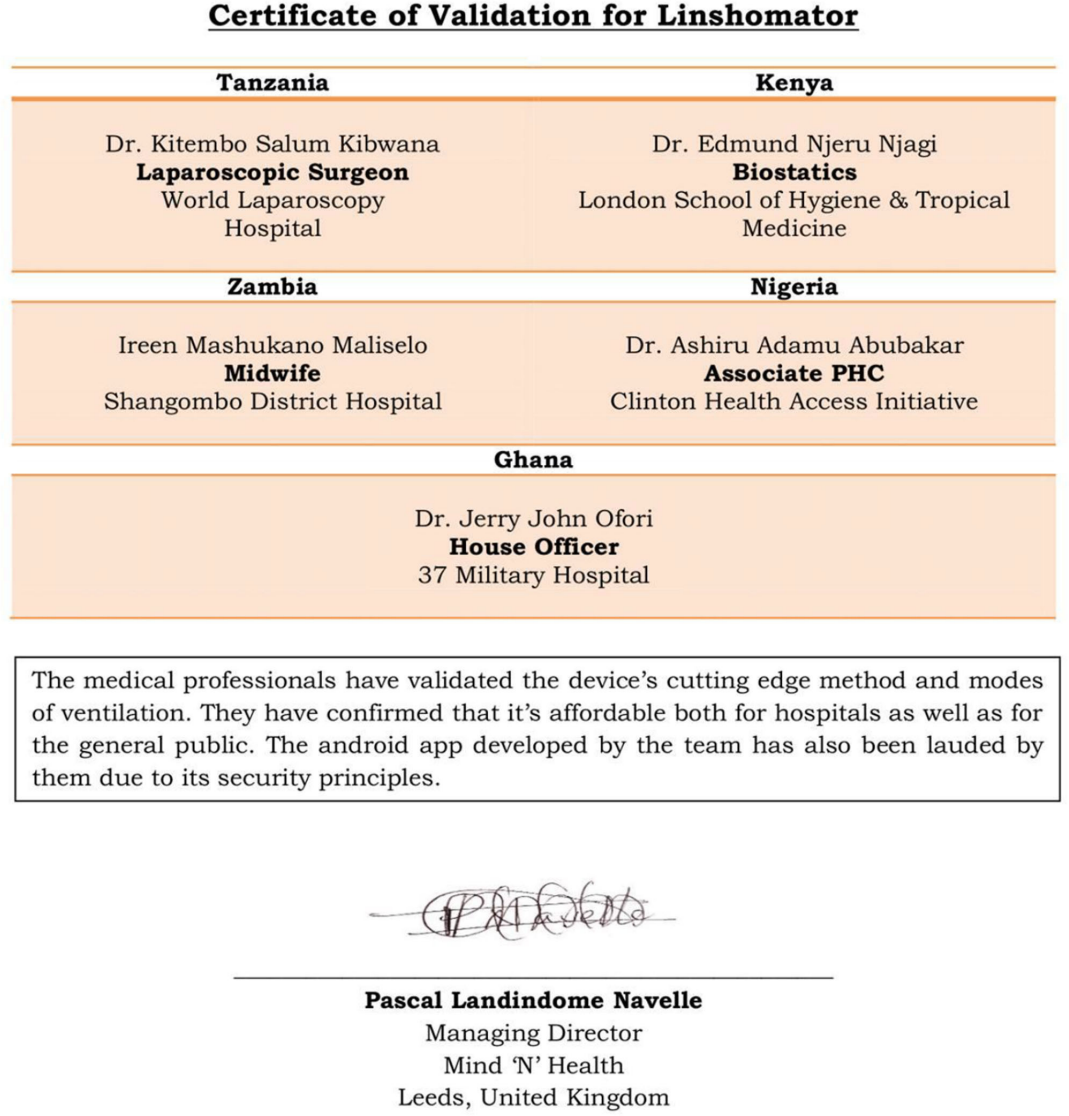
Validation for the automation of the device received from different medical professionals across Africa.

## Conclusion

The design of the mechanical ventilation system and automation layout has been presented in this paper. The ventilation system was designed in such a way that it would be able to provide fresh air up to 1.05 L per push/stroke as per patient's optimal requirements. The variation in the air discharge is however achieved with the integrated automation system in accordance with the selected mode of ventilation. The pulse rate and the breathing rate of the patient would be displayed in the screen and integrated app with the help of a pulse-oximeter sensor. Besides, the SpO_2_ rate will also be displayed in the system to monitor oxygen requirements. Most importantly, a multi-fold alarm system adhering to all kinds of potential failure was also integrated for the safety of the patients. An integrated Android app will add more credibility and usability to the system. There are still provisions for minor improvements that can be undertaken for further research such as adding more ventilation modes into the system and using machine learning to control the medical parameters involved in ventilation.

## Data Availability Statement

The original contributions presented in the study are included in the article/Supplementary Materials, further inquiries can be directed to the corresponding author/s.

## Ethics Statement

The studies involving human participants were reviewed and approved by World Laparoscopy Hospital. Written informed consent to participate in this study was provided by the participants' legal guardian/next of kin.

## Author Contributions

All authors listed have made a substantial, direct and intellectual contribution to the work and approved it for publication.

## Conflict of Interest

The authors declare that the research was conducted in the absence of any commercial or financial relationships that could be construed as a potential conflict of interest.
